# Feasibility of dynamic T_2_ *‐based oxygen‐enhanced lung MRI at 3T

**DOI:** 10.1002/mrm.29914

**Published:** 2023-11-27

**Authors:** Mina Kim, Josephine H. Naish, Sarah H. Needleman, Marta Tibiletti, Yohn Taylor, James P. B. O'Connor, Geoff J. M. Parker

**Affiliations:** ^1^ Department of Medical Physics and Biomedical Engineering, Centre for Medical Image Computing (CMIC) University College London London UK; ^2^ Bioxydyn Limited Manchester UK; ^3^ BHF Manchester Centre for Heart and Lung Magnetic Resonance Research (MCMR) Manchester University NHS Foundation Trust Manchester UK; ^4^ Division of Cancer Sciences University of Manchester Manchester UK; ^5^ Division of Radiotherapy and Imaging Institute of Cancer Research London UK

**Keywords:** 3T, dynamic, free‐breathing, lung, OE‐MRI, oxygen‐enhanced MRI, repeatability, reproducibility

## Abstract

**Purpose:**

To demonstrate proof‐of‐concept of a T_2_*‐sensitized oxygen‐enhanced MRI (OE‐MRI) method at 3T by assessing signal characteristics, repeatability, and reproducibility of dynamic lung OE‐MRI metrics in healthy volunteers.

**Methods:**

We performed sequence‐specific simulations for protocol optimisation and acquired free‐breathing OE‐MRI data from 16 healthy subjects using a dual‐echo RF‐spoiled gradient echo approach at 3T across two institutions. Non‐linear registration and tissue density correction were applied. Derived metrics included percent signal enhancement (PSE), ∆R_2_* and wash‐in time normalized for breathing rate (τ‐nBR). Inter‐scanner reproducibility and intra‐scanner repeatability were evaluated using intra‐class correlation coefficient (ICC), repeatability coefficient, reproducibility coefficient, and Bland–Altman analysis.

**Results:**

Simulations and experimental data show negative contrast upon oxygen inhalation, due to substantial dominance of ∆R_2_* at TE > 0.2 ms. Density correction improved signal fluctuations. Density‐corrected mean PSE values, aligned with simulations, display TE‐dependence, and an anterior‐to‐posterior PSE reduction trend at TE_1_. ∆R_2_* maps exhibit spatial heterogeneity in oxygen delivery, featuring anterior‐to‐posterior R_2_* increase. Mean T_2_* values across 32 scans were 0.68 and 0.62 ms for pre‐ and post‐O_2_ inhalation, respectively. Excellent or good agreement emerged from all intra‐, inter‐scanner and inter‐rater variability tests for PSE and ∆R_2_*. However, ICC values for τ‐nBR demonstrated limited agreement between repeated measures.

**Conclusion:**

Our results demonstrate the feasibility of a T_2_*‐weighted method utilizing a dual‐echo RF‐spoiled gradient echo approach, simultaneously capturing PSE, ∆R_2_* changes, and oxygen wash‐in during free‐breathing. The excellent or good repeatability and reproducibility on intra‐ and inter‐scanner PSE and ∆R_2_* suggest potential utility in multi‐center clinical applications.

## 1 INTRODUCTION

Oxygen‐enhanced MRI (OE‐MRI) is a method that has been demonstrated for imaging lung function.^1^, ^2^ To date, the majority of OE‐MRI studies have made use of T_1_‐weighted acquisitions, which enable regional investigation of oxygen delivery to the tissues and blood pool via ventilation and gas exchange across the alveolar epithelium into the bloodstream, since a change in T_1_ occurs due to the paramagnetic nature of oxygen dissolved in the parenchyma. During the past two decades, investigators have shown promising OE‐MRI results for evaluating abnormal lung function in patients with chronic lung disease including interstitial pneumonia, pulmonary emphysema, cystic fibrosis, pulmonary thromboembolism, chronic obstructive pulmonary disease (COPD), lung cancer, and asthma.^3^, ^4^, ^5^, ^6^, ^7^, ^8^, ^9^

To date, most OE‐MRI studies in lungs have been performed at field strengths of 1.5T or lower,^10^ while there is a scarcity of literature on OE‐MRI methods at 3T. Previous studies at 3T used T_1_‐weighted single‐slice non‐selective inversion‐recovery half‐Fourier acquisition single‐shot turbo spin echo (HASTE),^11^ 3D radial UTE pulse sequence with HASTE acquisition,^12^ and 3D T_1_‐weighted fast‐field echo (FFE).^13^ While all these methods used separate free‐breathing acquisitions at 21% and 100% O_2_, methodologies for free‐breathing lung OE‐MRI over entire time course enabling dynamic parametrisation have not been established at 3T.

There are inherent difficulties in conducting lung MRI at 3T. The magnetic susceptibility differences at the numerous air–tissue interfaces within the lung are greater than at lower field strengths and significantly shorten T_2_* in the parenchyma, thereby reducing the signal available for gradient echo‐based methods. Additionally, T_1_ relaxivity of oxygen decreases with increased field strength,^14^ further diminishing the sensitivity of the commonly‐used ∆R_1_‐based OE‐MRI methods. Moreover, when employing gradient echo‐based methods, the competing ∆R_2_* effect becomes substantial and dominates over the ∆R_1_ effect at 3T, even at short TE.^15^ Nevertheless, spoiled gradient echo pulse sequences are the most widely used methods for dynamic MRI data collection, enabling rapid acquisition of images with good spatial coverage and resolution. Although spin echo‐based and ultrafast echo‐based methods are not compromised by T_2_* effects, they may be limited to T_1_‐sensitized “static” (e.g., breath‐hold or gated) OE‐MRI due to relatively low temporal resolution.

Given the increasing clinical availability of 3T MRI, the above technical challenges underscore the necessity for novel methodological advancements, aiming to facilitate the widespread adoption of dynamic OE‐MRI at 3T. We hypothesized that T_2_*‐sensitized dynamic OE‐MRI, characterized by a dual‐echo acquisition, can enhance the sensitivity of lung signal detection and this work is motivated by a need to evaluate the performance of our proposed method. Furthermore, in order for OE‐MRI to find application in clinical research and, ultimately, clinical practice, it is important to harmonize protocols across centers and vendors. Additionally, any derived biomarkers must exhibit satisfactory levels of repeatability and reproducibility.^16^

The primary objective of the present study was to demonstrate proof‐of‐concept of the T_2_*‐sensitized method and an initial assessment of its robustness. Specifically, we aimed (1) to use simulations to characterize the OE‐MRI signal across a range of achievable sequence parameters at 3T; (2) to evaluate the feasibility of the T_2_*‐sensitized OE‐MRI method at 3T in healthy volunteers; and (3) to assess the repeatability and reproducibility of the dynamic OE‐MRI metrics in healthy volunteers across two sites and two vendors.

## 2 METHODS

For dynamic multi‐slice OE‐MRI acquisition, we implemented a dual‐echo RF‐spoiled gradient echo sequence to enable estimation of T_2_*. We aimed to obtain images with a high temporal resolution to minimize motion artifact during free‐breathing while maximizing lung coverage and enabling reasonable spatial resolution. We determined that TR = 16 ms and matrix size = 96 × 96, would enable dynamic temporal resolution <2 s and acquisition of six slices. Both TEs for the dual‐echo acquisition should be as short as possible to avoid losing signal due to low T_2_*, and flip angle (FA) should be chosen to maximize signal difference between normoxia and hyperoxia. The human data experimental workflow is outlined in Figure S1 (Supporting Information).

### 2.1 Simulations

We simulated the signal behavior of the dual‐echo RF‐spoiled gradient echo sequence at our chosen TR (16 ms) over a range of FA and TE values to match the experimental sequence and protocol, described as follows.^17^ First, the expected signal difference between air breathing and 100% oxygen breathing (∆S) and percent signal enhancement (PSE; 100% × ∆S/S(air)) in the lung was simulated as a function of FA up to 30° and TE up to 3 ms using the following parameters: T_1_ (air) = 1281 ms,^18^ T_1_ (100% O_2_) = 1102 ms,^18^ T_2_* (air) = 0.68 ms, and T_2_* (100% O_2_) = 0.62 ms. Given the absence of previously reported lung hyperoxic T_2_* values in the literature, we computed T_2_* values for all subjects in our study, then averaged those values for use in the simulations (Table S1). Then, the expected ∆S values were simulated as a function of TE using FA of 5°, which was shown to maximize the absolute signal difference between the 21% and 100% oxygen images. Simulations were performed in MATLAB R2022b (MathWorks, Natick, MA).

### 2.2 Participants

Following local research ethics committee approval (Ref: 18837/001) and written informed consent, we recruited 16 healthy volunteers with no previous record of lung diseases. Of these, eight subjects (three males, age range = 26–54 y, median = 39.5 y) underwent lung MRI on a 3T scanner from two vendors in different cities (Philips Ingenia in London, and Siemens MAGNETOM Vida in Manchester, UK) at a 4‐wk interval. Eight separate subjects (four males, age range = 23–51 y, median = 27) were scanned twice to assess scan‐rescan repeatability at a 4‐ to 6‐wk interval using the Philips scanner. The inclusion criteria for participants specified healthy adult volunteers aged 18–80 y, who self‐declared absence of current or previous lung diseases and exhibited no MRI contraindications.

### 2.3 MRI acquisition

**FIGURE 1:**
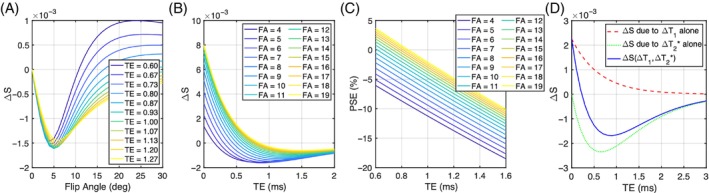
(A) The predicted OE signal change ∆S plotted as a function of flip angle at multiple TE, with TR = 16 ms. (B) The predicted signal change ∆S plotted as a function of TE with multiple flip angle, with TR = 16 ms. (C) PSE plotted as a function of TE at multiple flip angles, with TR = 16 ms. (D) The expected OE signal change for the T_1_‐weighted RF spoiled gradient echo acquisition at 3T due to ∆T_1_ alone (red dashed line), ∆T_2_* alone (green dotted line), and both (blue solid line), assuming literature‐reported values for T_1_ and measured T_2_* in the lungs at 21% oxygen and 100% oxygen, flip angle = 5°, and TR = 16 ms.

Where possible, protocols for the two different scanner manufacturers utilized identical acquisition parameters for a 2D interleaved multi‐slices dual‐echo RF‐spoiled gradient echo sequence, while some options required manufacturer‐specific parameters. Site‐independent parameters included: six coronal slices of 10 mm thickness with 4 mm gap with phase‐encoding right/left; in‐plane resolution 4.69 × 4.69 mm^2^; FOV covering entire lungs in all 16 volunteers (except for the inter‐slice gaps in the anterior/posterior direction); TR = 16 ms; matrix size = 96 × 96; and dynamic temporal resolution = 1.54 s. Selection of FA (5°) was based on our simulations (Figure 1). The shortest TE values available for the chosen acquisition were selected for each scanner (L, Philips in London; M, Siemens in Manchester): TE_1L_ = 0.71 ms, TE_2L_ = 1.2 ms; TE_1M_ = 0.81 ms, TE_2M_ = 1.51 ms. Scan parameters for each vendor are listed in Table 1.

**TABLE 1:** Scan parameters.

	Scanner in London	Scanner in Manchester
Manufacturer	Philips	Siemens
Model	Ingenia	MAGNETOM Vida
Field strength (T)	3.0	2.9
RF coil used	32‐channel torso coil in combination with the posterior coil	18‐channel body coil in combination with the 32‐channel spine coil
Max. gradient strength (mT/m)	45	45
Max. slew rate (T/m/s)	200	200
TR (ms)	16	16
Echoes	Full	Half
Minimum achievable TE_1_ (ms)	0.71	0.81
Minimum achievable TE_2_ (ms)	1.2	1.51
FOV (mm × mm)	450 × 450	450 × 450
No. of slices	6	6
Slice thickness (mm)	10	10
Gap (mm)	4	4
Acquired matrix	96 × 96	96 × 96
Orientation	Coronal	Coronal
Pixel size (mm × mm)	4.7 × 4.7	4.7 × 4.7
Flip angle (°)	5	5
Bandwidth (Hz/Px)	4488	2000
Parallel imaging	N	N
NSA	1	1
Time resolution (s)	1.54	1.54
Number of dynamics	340	340

Subjects were fitted with a disposable/MRI‐compatible non‐rebreathing mask (Intersurgical, Berkshire, UK) to allow for medical air and 100% oxygen delivery while lying supine in the scanner. Piped gases were delivered to the subject at 15 L/min using a standard low flow oxygen blender (Inspiration Healthcare, Leicestershire, UK). The initial 60 dynamic acquisitions were obtained while breathing medical air. The gas supply was then switched to 100% O_2_ for the following 150 dynamic acquisitions, after which the supply was returned to medical air for further 130 acquisitions. Images were acquired during uncontrolled free‐breathing to minimize participant burden and avoid interrupting gas delivery. Total scanning time for the dynamic series was approximately 9 min.

### 2.4 Data analysis

For motion correction, non‐linear image registration was performed on the dynamic time series data using Advanced Normalization Tools (ANTs).^19^, ^20^ Subsequently, the lung parenchyma, excluding central major vasculature, was manually segmented from registered images. For an initial exploration of the data, first, image registration and density correction were performed as described below. Secondly, averaged hyperoxia images (61st to 210th) were subtracted from averaged normoxia images (10th to 60th). Last, mean PSE maps was calculated from the subtracted images normalized to the averaged normoxia images.

For our main data analysis, the dynamic series were fitted using exponential functions to characterize oxygen wash‐in (encompassing the downslope between the plateau regions of the curve) and wash‐out (the upslope and return to baseline). The baseline for the exponential fit was defined as the averaged signal intensity across all normoxia time points before O_2_ inhalation as described in Eq. 1. The curve was fitted with the two functional forms described in Eq. 2 for the downslope and Eq. 3 for the upslope,

(1) $$A_{1}\left(x\right)=\frac{\sum S\left(t,x\right)}{\mathrm{tp}1},1\leq t\leq \mathrm{tp}1$$

(2) $$S\left(t,x\right)=\left[A_{1}\left(x\right)\text{–}A_{2}\left(x\right)\right]\cdot e^{-t/\tau \left(x\right)}+A_{2}\left(x\right),\mathrm{tp}1+1\leq t\leq \mathrm{tp}2$$

(3) $$S\left(t,x\right)=\left[A_{1}\left(x\right)\text{–}A_{2}\left(x\right)\right]\cdot \left(1-e^{-t/\tau \left(x\right)}\right)+A_{2}\left(x\right),\mathrm{tp}2+1\leq t$$

where *A*_1_(**x**) and *A*_2_(**x**) are the baseline and fitted negative maximum hyperoxia intensity (or plateau value) at position **x**, respectively, and τ, tp1, and tp2 are the fitted wash‐in time and the provided gas switching time points (i.e., tp1, air to O_2_; tp2, O_2_ to air). Maximum PSE maps were produced by the subtraction of the baseline from the negative maximum hyperoxia value (*A*_1_–*A*_2_), normalized to the baseline *A*_1_. We additionally defined a breathing rate‐normalized wash‐in time, τ‐nBR as the product of τ and the average breathing rate over the dynamic series.

As differences in lung tissue density can influence the measured signal enhancement between normoxia and hyperoxia, time‐varying PSE maps were calculated twice, with and without a voxel‐wise tissue density correction. Uncorrected PSE values were calculated by the subtraction of normoxia signal (S_21_) from hyperoxia signal (S_100_), normalized to S_21_ as.

(4) $$\mathrm{PSE}\text{\_}u\left(t,x\right)=\frac{S_{100}\left(t,x\right)-S_{21}\left(x\right)}{S_{21}\left(x\right)}\times 100\%.$$

Tissue density variation was corrected using the adapted sponge model.^21^, ^22^, ^23^, ^24^ The whole‐lung fractional volume change V was calculated at each time point by averaging the Jacobian determinant from the registration over all voxels in the lung mask across all slices. The Jacobian determinant was only used to obtain an estimate of lung volume change, with density correction based on the signal intensity variation associated with the lung volume change as described further below. The respiratory index α_local_ was estimated voxel‐wise (locally at the position **x**) by linear regression estimation of the observed signal intensity S as a function of V as

(5) $$\alpha_{\text{local}}\left(t,x\right)=-\frac{\partial (\log\left(S\left(t,x\right)\right)}{\partial \left(\log\left(V\left(t,x\right)\right)\right)}.$$

Then, the α_local_ values were applied as a voxel‐wise density correction as

(6) $$S_{C}\left(t,x\right)=S\left(t,x\right)V{\left(t,x\right)}^{-\alpha_{\text{local}}\left(t,x\right)}$$

where S_C_(t,x) represent the corrected S(t,x). Corrected PSE values were quantified as

(7) $$\mathrm{PSE}\text{\_}c\left(t,x\right)=\frac{S_{100c}\left(t,x\right)-S_{21c}}{S_{21c}}\times 100\%$$

where S_100c_(*t* ,x) and S_21c_(*t* ,x) represent the corrected S_100_(*t* ,x) and S_21_(*t* ,x), respectively. To compare pre‐ and post‐density correction, we calculated median PSE values within masks at each TE twice, either across all six slices or the two most posterior slices, excluding anterior slices with poor SNR. The median PSE value for each slice was then averaged across all subjects. For intra‐scanner repeatability, median PSE values were averaged over all six slices at each TE.

The R_2_* of each voxel was quantified analytically from the magnitude‐reconstructed signal from the masked lung images acquired at TE_1_ and TE_2_ after tissue density correction as described in Eqs. 5 and 6. ∆R_2_* maps were calculated by the subtraction of mean normoxia R_2_* maps across multiple time points (30th to 60th time series acquisitions) from mean hyperoxia R_2_* maps across multiple time points (120th to 180th). Median ∆R_2_* values were averaged over the two most posterior slices for multi‐site comparison between Manchester and London, and six slices for scan‐rescan comparison in London.

Data were analyzed by an experienced (>10 years) MRI physicist using a computational pipeline written in MATLAB R2022b (MathWorks, Natick, MA), taking ˜60 min per subject visit, primarily due to motion correction.

### 2.5 Statistical analysis

Normality was assessed for all metrics using the Shapiro–Wilk test. For non‐normally distributed metrics, we log‐transformed the data before statistical analyzes (Figure S1). We used Bland–Altman plots with 95% limits of agreement (LOA) and derived the repeatability coefficient (RC), reproducibility coefficient (RDC), and intraclass correlation coefficient (ICC) to evaluate the agreement of repeated measures, as recommended in the QIBA guidelines.^25^ For the log‐transformed metric, we calculated the asymmetric cut‐points for RC and RDC through back‐transformation,^26^ and ICC values were computed as described in Pleil et al.^27^ An inter‐rater ICC analysis was conducted on London data from eight volunteers at the initial time point, using additional lung masks outlined by a second rater. The agreement levels were: excellent for ICC > 0.74, good for ICC 0.6–0.74, fair for ICC 0.4–0.59, and poor for ICC < 0.4.^28^ Coefficient of variation (CV) was calculated across all subjects at each TE. All statistical analyzes were performed using SPSS v28.0 (SPSS Inc, Chicago, IL).

## 3 RESULTS

### 3.1 The effect of oxygen on signal intensity–simulations

Simulations show that ∆T_2_*‐induced negative enhancement (∆S) for our chosen TR = 16 ms is maximum at FA ˜5°, independent of the choice of TE (Figure 1A). The amplitude of the TE dependence of ∆S reduces with smaller FA (Figure 1B), with negative‐going signal change occurring at shorter TE. The magnitude of negative PSE increases with lower FA and longer TE, while PSE values are closer to 0 at shorter TE and high FA (Figure 1C). We also observed that ∆T_2_* dominates the signal change and produces negative contrast at TE > 0.2 ms for FA = 5° (Figure 1D). The expected signal change at TE_1L_ (0.71 ms) is about 55% more sensitive to changes in ∆T_1_ and about 21% more sensitive to changes in ∆T_2_* than at TE_2L_ (1.2 ms) (Figure 1D).

### 3.2 The effect of oxygen on signal intensity–experimental

**FIGURE 2:**
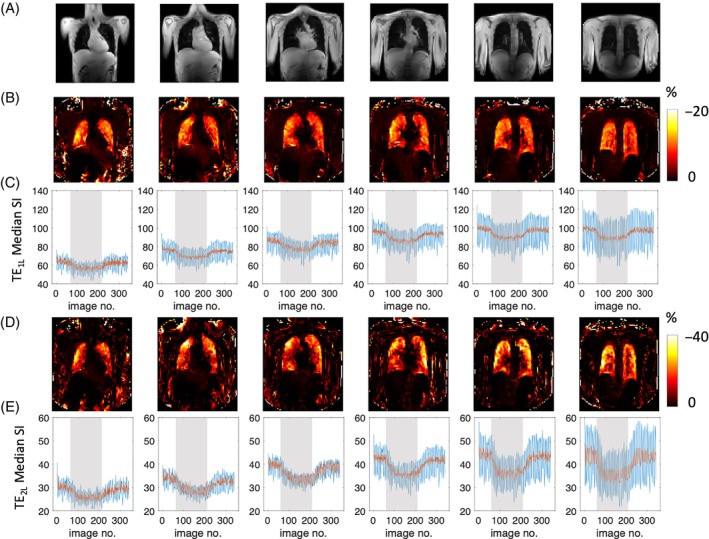
(A) The typical location of the six slices from anterior to posterior. Example subject data show unmasked percentage signal change maps obtained with (B) TE_1L_ (0.71 ms) and (D) TE_2L_ (1.2 ms), and (C, E) the corresponding time course curves of the median signal intensity from masked, registered lung for each slice. Blue lines show uncorrected signal; red line shows signal after density correction.

Typical location of the acquired images is shown in Figure 2A. PSE maps at both TEs demonstrate uniform PSE across the parenchyma (Figure 2B,D). As expected, due to the dominant effect of T_2_* changes, time course plots of mean PSE from masked lungs exhibit negative contrast induced by 100% O_2_ inhalation (Figure 2C,E), in agreement with the simulated results (Figure 1D). The median signal intensity is higher in posterior slices due to greater proton density, associated with the subjects' supine position (Figures 2C,E and S2).

### 3.3 The effect of density correction

The time course plots (Figure 2C,E) post‐density correction (red solid line) show smaller magnitude signal fluctuation than pre‐density correction (blue solid line) due to the reduced impact of respiratory motion‐induced signal changes. Example signal time courses with their downslope and upslope fits (Eqs. 2 and 3) also show improvement in time course wash‐in fitting with tissue density correction (Figure S3).

Table 2 summarizes the mean PSE and CV before and after applying density correction for the evaluation of inter‐scanner TE‐dependence. Across eight healthy volunteers, the magnitudes of all negative PSE values were reduced by applying tissue density correction. Moreover, this correction enhanced the linearity of the reduction in negative PSE magnitudes. In addition, CV of mean PSE was also decreased for all except TE_1M_ PSE (mean PSE values over two posterior slices) or TE_2M_ PSE (mean PSE values over all six slices). All repeatability metrics were improved by the density correction step (Figure S4) as also described in the Section 3.7.

**TABLE 2:** Inter‐scanner TE‐dependence assessment of uncorrected and corrected mean (±SD) PSE measurements from eight traveling healthy volunteers in four different TEs (TE_1L_ and TE_2L_ in London; TE_1M_ and TE_2M_ in Manchester).

		TE (ms)	Averaged over two posterior slices		Averaged over all six slices	
			Mean PSE (%) ± SD (%)	CV (%)	Mean PSE (%) ± SD (%)	CV (%)
Uncorrected PSE mapping (Eq. 4)	TE_1L_ PSE	0.71	−9.61 ± 4.24	44.13	−9.40 ± 2.28	24.28
	TE_1M_ PSE	0.81	−8.61 ± 1.97	22.84	−9.68 ± 1.69	17.41
	TE_2L_ PSE	1.20	−14.87 ± 4.51	30.35	−14.98 ± 3.31	22.12
	TE_2M_ PSE	1.51	−14.01 ± 2.77	19.80	−11.43 ± 1.53	13.40
Corrected PSE mapping (Eq. 7)	TE_1L_ PSE	0.71	−6.55 ± 1.92	29.32	−6.81 ± 1.47	21.62
	TE_1M_ PSE	0.81	−8.06 ± 1.84	22.85	−9.46 ± 1.82	19.28
	TE_2L_ PSE	1.20	−12.20 ± 2.75	22.56	−11.92 ± 1.78	14.98
	TE_2M_ PSE	1.51	−13.37 ± 2.46	18.39	−11.14 ± 1.73	15.55

*Note*: The mean PSE and CV were calculated across all subjects at each TE based on the measurement from the two most posterior slices and all six slices.

### 3.4 TE dependence

While the mean signal intensity is higher at TE_1L_ than for TE_2L_ (Figure 2C,E), PSE is greater at TE_2L_ than at TE_1L_ (Figure 2B,D), again in agreement with our simulations (Figure 1B,C). The TE dependence of PSE expected from simulations (Figure 1C) is also observed in the density‐corrected PSE values from four separate TEs at two sites (Table 2).

### 3.5 ∆R_2_ * quantification

**FIGURE 3:**
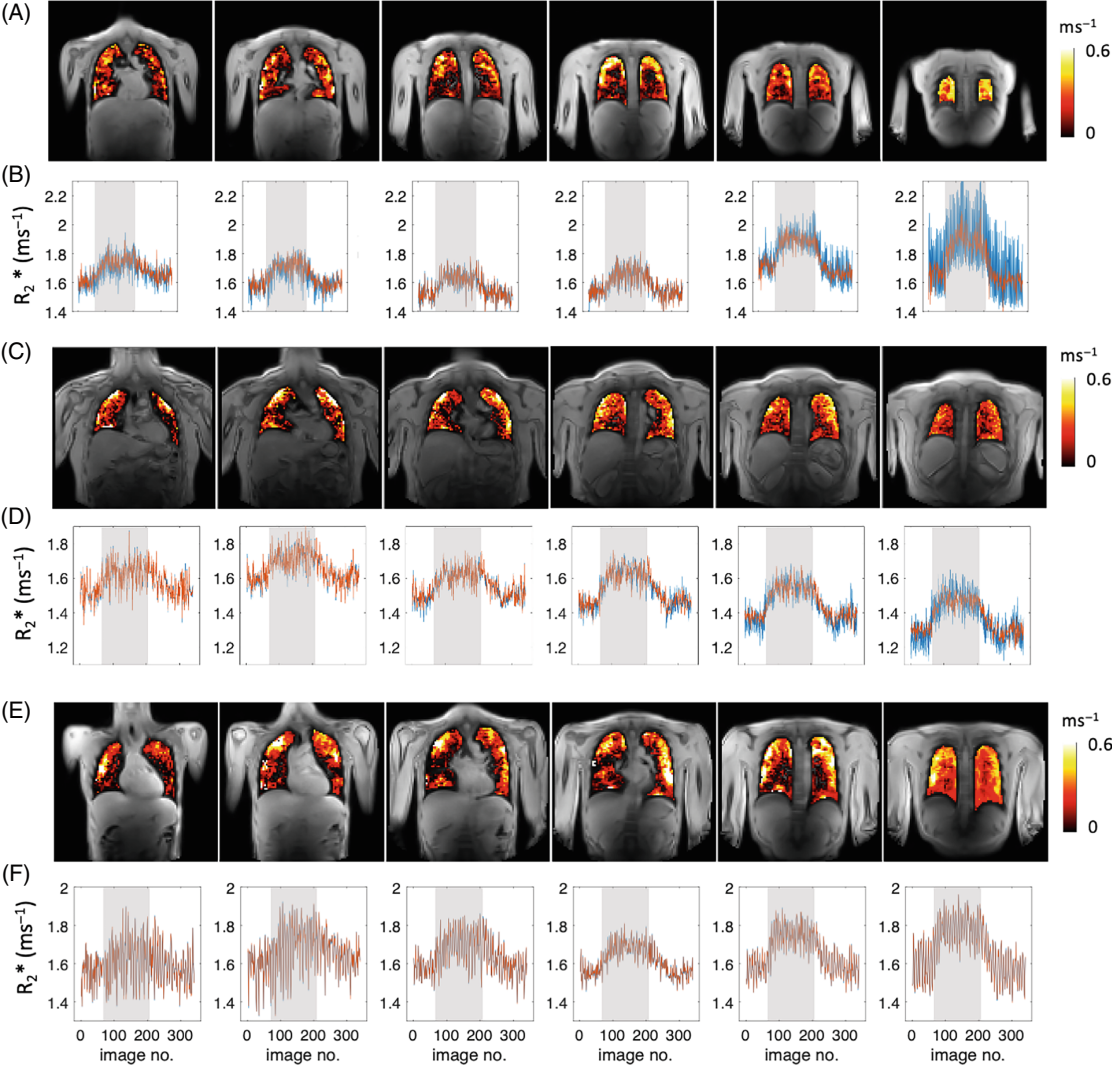
Examples of three subjects: (A, C, E) the plateau ∆R_2_* maps of masked lung, six slices from anterior to posterior and (B, D, F) the corresponding time course curves of median R_2_* from masked lung along for each slice. Increase of R_2_* due to 100% O_2_ inhalation is visible in all slices but clearer in posterior slices.

Figure 3 shows examples of plateau ∆R_2_* maps across six slices from anterior to posterior, the corresponding time course plots of the median R_2_* from the maps of masked lungs for each slice. Median ∆R_2_* maps illustrate clear O_2_ delivery in the entire lung (Figure 3A,C,E), with a spatial distribution that is heterogeneous compared to the patterns observed in the PSE maps (Figure 2).

Median R_2_* time course plots show R_2_* is largely unaffected by density correction (because the calculation of R_2_* normalizes for density) except for the last posterior slice. We observed that the discrepancy between tissue density corrected (red) and uncorrected (blue) ∆R_2_* plots (Figure 3B) is a common occurrence among volunteers with smaller lung volumes. In such cases, the final posterior slice is aligned with the rear of the lungs, adjacent to the ribcage (Figure 3A) and appears to be influenced by the partial volume effects.

**FIGURE 4:**
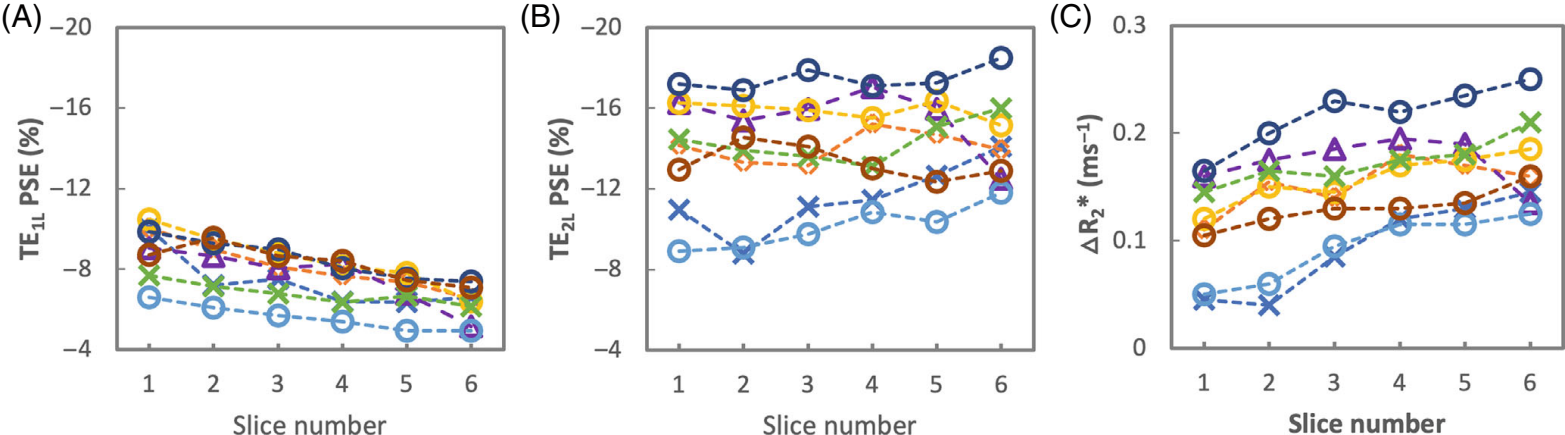
The PSE from masked, registered, tissue density corrected lung for each slice of eight individual subjects scanned in London with (A) TE_1L_ (0.71 ms) and (B) TE_2L_ (1.2 ms). (C) Equivalent plot for ∆R_2_*.

A trend of ∆R_2_* increase from anterior to posterior slices is observed (Figure 4C), and the mean T_2_* values of 16 healthy volunteers from the two posterior slices were 0.68 ± 0.05 ms (T_2_* for 21% O_2_) and 0.62 ± 0.05 ms (T_2_* for 100% O_2_), while mean ∆R_2_* was 0.14 ± 0.03 ms^−1^ (Table 3).

**TABLE 3:** Repeatability and reproducibility for all metrics.

	Mean ± SD	Difference		95% LOA		RC	RDC	ICC_intra_	ICC_inter_	ICC_inter‐rater_
		Intra‐scanner	Inter‐scanner	Intra‐scanner	Inter‐scanner					
TE_1L_ PSE^a^	−7.59 ± 1.60	−0.51	‐	(−2.36, 1.33)	‐	2.61	‐	0.77	‐	0.99
TE_2L_ PSE^a^	−13.98 ± 2.66	−0.57	‐	(−2.99, 1.84)	‐	3.42	‐	0.92	‐	0.99
△R_2_*^b^	0.14 ± 0.03	0.00	0.00	(−0.05, 0.04)	(−0.06, 0.06)	0.06	0.11	0.94	0.70	0.99
Log‐transformed TE_1_ τ‐nBR^c^	0.75 ± 0.18	‐	‐	‐	‐	(−0.66, 1.96)	(−0.69, 2.22)	0.28	0.45	0.99
Log‐transformed TE_2_ τ‐nBR^c^	0.81 ± 0.16	‐	‐	‐	‐	(−0.56, 1.27)	(−0.58, 1.40)	0.28	0.54	0.99

*Note*: First column: mean ± SD of each metric. PSE values were computed from the two repeated sessions in London (16 data sets in total) while △R_2_*, and wash‐in time‐normalized for breathing rate (τ‐nBR) were computed from traveling volunteers at both sites and the two repeated sessions in London (32 data sets in total). The mean value for each metric was averaged across all six slices except that for △R_2_* which was averaged across two posterior slices. Middle columns: mean difference between two sessions, the Bland–Altman 95% LOA for inter‐ and intra‐scanner comparisons, RC for intra‐scanner comparisons, and RDC for inter‐scanner comparisons. Last three columns: ICC for intra‐scanner variation (ICC_intra_), inter‐scanner variation (ICC_inter_), and inter‐rater variation (ICC_inter‐rater_) based on absolute agreement, two‐way mixed‐effects model. Notably, the ICC_inter‐rater_ values exceeded 0.99 for all metrics. The excellent ICC_inter‐rater_ values were expected, as the only manual step is lung segmentation, and the median voxel value for all reported measurements is largely insensitive to differences in lung outlining.

^a^ Unit for PSE and associated RC and RDC: %.

^b^ Unit for △R_2_* and associated RD and RDC: ms^−1^.

^c^ Unitless for log‐transformed τ‐nBR and associated RC and RDC.

### 3.6 Signal variation with slice position in the lung

The PSE of TE_1L_ gradually decreases from anterior to posterior slices across all subjects (Figure 4A), whereas the PSE of TE_2L_ do not noticeably change (Figure 4B). ∆R_2_* shows a gradual increase from anterior to posterior.

### 3.7 Repeatability and reproducibility

All metrics were normally distributed except a subset of τ‐nBR (Table S2).

**FIGURE 5:**
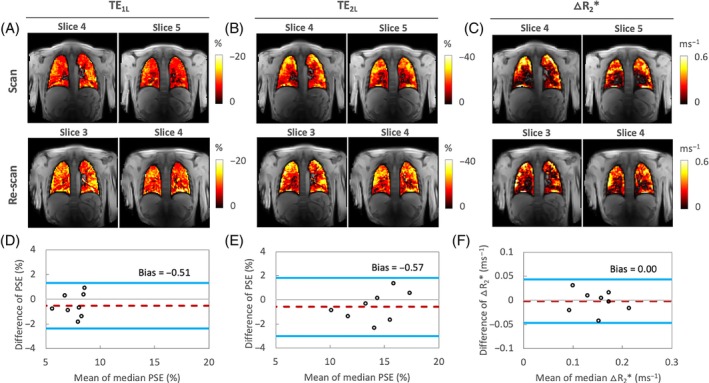
Example of one subject and Bland–Altman analysis comparing PSE and △R_2_* between two separate sessions (repeatability) in London. (A) mean PSE with TE = 0.71 ms, (B) mean PSE with TE = 1.2 ms, (C) mean △R_2_*, and (D, E) Bland–Altman plots for the repeated measurements of PSE from the first and second TE and (F) △R_2_* (intra‐scanner, intra‐subject). (A, B) The mean PSE values from eight healthy volunteers varied little between the repeat scans (−7.39% ± 1.61% and −7.79% ± 1.58% at TE_1L_; −13.71% ± 2.96% and −14.27% ± 2.31% at TE_2L_). (D–F) The data points correspond to individual participants for difference between two visits to London site.

Example intra‐scanner PSE maps show relatively homogeneous enhancement at both TEs (Figure 5A,B). The mean PSE values from eight healthy volunteers varied little between the repeat scans.

The plateau △R_2_* maps from the same data set show relatively heterogeneous △R_2_* distribution, wherein certain structures, particularly areas of major vasculature, do not appear to respond to 100% O_2_ inhalation (Figure 5C). This is expected as the R_2_* change is mainly due to gaseous oxygen in the alveoli but not dissolved oxygen as previously reported.^15^

The Bland–Altman plot analyzes of the repeated measurements of PSE and △R_2_* indicate little and insignificant bias between two intra‐scanner measurements in London, respectively, as shown by the 95% LOA (Figure 5D–F). The ICC and RC measurements of PSE at TE_1L_ and TE_2L_, and intra‐scanner △R_2_* show excellent intra‐scanner repeatability (Table 3).

**FIGURE 6:**
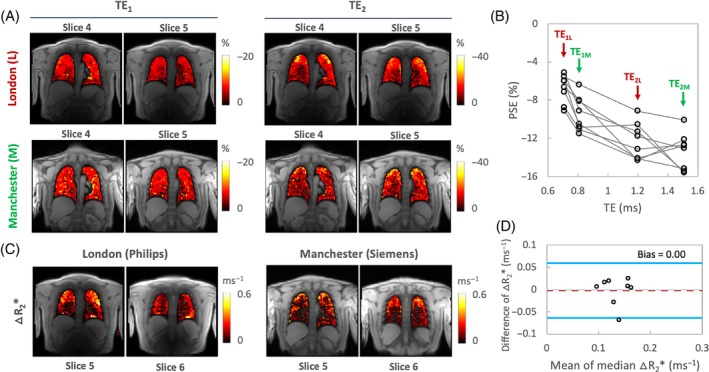
(A) An example of inter‐scanner intra‐subject reproducibility of PSE at TE_1_ and TE_2_ scanned using two MRI systems at two sites (London and Manchester). (B) PSE obtained at four separate TEs (TE_1L_ = 0.71 ms, TE_2L_ = 1.2 ms in London and TE_1M_ = 0.81 ms, TE_2M_ = 1.51 ms in Manchester). The combined PSE values from two MRI systems show a similar trend as a function of TE as the PSE simulation (Figure 1C). (C) Inter‐scanner intra‐subject reproducibility of △R_2_* from the same subject. (D) Bland–Altman analysis comparing △R_2_* between two scanners for the same subjects.

The PSE maps from inter‐scanner traveling volunteers' scans show similar spatial distribution of enhancement at TE_1_. However, the PSE observed at TE_2_ from the Manchester site exhibits more pronounced noise, which could be attributed to the signal approaching the noise floor at the longer TE (Figure 6A). Plots of the combined PSE values at four separate TEs from the two MRI systems display the expected TE dependence of the signal (Figure 6B, Table 2), similar to simulation (Figure 1C).

The △R_2_* maps display a lack of enhancement near major vasculature (Figure 6C). Notably, the △R_2_* maps obtained from the Manchester site continue to show increased noise, a pattern consistent with the TE_2M_ PSE maps. Nevertheless, △R_2_* comparison between two different scanners again shows little evidence of bias, with the 95% LOA measurements being (−0.06%, 0.06%) (Figure 6D). The values of ICC and RDC observed for inter‐scanner △R_2_* comparisons reflect good reproducibility (Table 2).

The ICC_inter_ measurement of the log‐transformed τ‐nBR values shows fair repeatability and reproducibility, while ICC_intra_, RC, and RDC values are in the poor range due to existence of outliers as shown in Figure S5.

## 4 DISCUSSION

In recent years, installations of a clinical 3T MR systems have significantly increased worldwide, often motivated by the higher SNR, relative to lower field systems. However, the viability of dynamic lung OE‐MRI at 3T has not to date been investigated. In this work, we demonstrate the feasibility of detecting dynamic OE signal change and quantifying ∆R_2_* due to oxygen breathing at 3T. To progress the translation of these biomarkers toward clinical use, we also evaluate the intra‐scanner repeatability and the inter‐scanner/cross‐site reproducibility of the proposed method. While a limited number of studies, to date, have demonstrated the feasibility of 3T T_1_‐weighted OE‐MRI,^11^, ^12^, ^13^ our present study is the first report to analyze detailed T_2_*‐weighted dynamic signal enhancement behavior, repeatability and reproducibility at 3T. Additionally, this investigation simultaneously entails the quantification of O_2_‐induced ∆R_2_*.

Our motivation for focussing on T_2_*‐related contrast at 3T is twofold. First, the longitudinal relaxivity of O_2_ is approximately 20% lower at 3T than 1.5T,^14^ leading to a proportionately smaller achievable ΔR_1_ at 3 T. Secondly, T_2_* in lung decreases with field strength, meaning that SNR in T_1_‐weighted OE‐MRI is much reduced. T_2_*‐based OE‐MRI has been proposed to counter some of these detrimental effects, although previously‐developed methods employed non‐standard acquisition methods.^29^ Of note, T_2_*‐related signal is potentially more specific to ventilation as it is expected to be an effect of changing concentrations of oxygen gas in the alveoli rather than dissolved oxygen.^15^ In the present study, we optimized a multi‐slice dual‐echo RF‐spoiled gradient echo acquisition; this method enables measurement of dynamic OE signal change at high temporal resolution with controllable T_2_*‐weighting, and monitoring of dynamic ∆R_2_*, simultaneously, while requiring no or minimal pulse programming. This easy implementation on standard clinical platforms is intended to assist in clinical translation of this technique.

### 4.1 T_2_ *‐weighting allows good oxygen delivery contrast at 3T

Our simulations (Figure 1D) show the expected dependence of spoiled gradient echo PSE on both ∆T_1_ and ∆T_2_*, which can lead to reduced oxygen‐related signal change if TE and FA are not optimized. Maximum (negative) PSE for our chosen TR of 16 ms is found with a FA of ˜5° across a wide range of TE (Figure 1A). Our simulations also indicate that negative PSE at TE longer than approximately 0.23 ms when using this FA, is due to the significant oxygen‐level dependent ∆R_2_* effect dominating the signal change in the lungs (Figure 1B,D). Our experimental data are consistent with our simulations, with negative PSE observed throughout the lung parenchyma, at levels that are in agreement with simulations. Importantly, this allows the generation of visually high‐quality mean PSE maps at the TEs used in this study (Figures 2, 5, 6). The mean PSE values of traveling healthy volunteers (Table 2) are consistent with the expected trend of PSE with variable TEs from our simulations (Figure 1C). Specifically, the simulated PSE plots, derived from the individual T_2_* values of eight healthy volunteers, demonstrate the significant impact of the chosen T_2_* values on the variability of simulated PSE values (Figure S6). Nonetheless, the experimental PSE values remain within the range of PSE values predicted by simulations.

The mean T_2_* value across all healthy volunteers for 21% O_2_ inhalation (0.68 ± 0.05 ms) aligns closely with the literature‐reported values (0.74 ± 0.1 ms) at 3T.^30^ We observed that upon 100% O_2_ inhalation, the mean T_2_* value decreased by about 9% relative to normoxia, resulting in ∆R_2_* of 0.14 ± 0.03 ms^−1^. To the best of our knowledge, this is the first study that reports mean values of hyperoxic T_2_* and ∆R_2_* of healthy human lungs at 3T. While the influence of T_2_* on PSE is large at 3T, the effect will also be present at lower field strengths when using gradient echo methods and should be accounted for when interpreting nominally T_1_‐weighted OE‐MRI.^17^

### 4.2 T_2_ *‐weighted OE‐MRI demonstrates good repeatability and reproducibility

The Bland–Altman, RC, and ICC analysis of the repeated measurements of PSE and △R_2_* suggest high intra‐scanner repeatability (Table 3, Figure 5). They also demonstrate that comparable dynamic OE‐MRI protocols for the lung can be implemented at 3T across different sites and scanners with good repeatability and reproducibility for △R_2_*. While maximum gradient strength and maximum gradient slew rate between the two systems from the different manufacturers are identical, matching TE and bandwidth between scanners proved challenging, which results in variability of OE signal enhancement. For this reason, direct reproducibility assessments for PSE were not feasible although the variation in PSE with TE between scanners closely aligned with our simulations (Figures 1C and 6B). We were able to assess ∆R_2_* reproducibility, as T_2_* signal decay is, to the best of our knowledge, monoexponential with TE in the lung. In this work, we derived a new metric, τ‐nBR, to compensate differences in individual participant's breathing patterns between scans, which have a direct impact on ventilation. The τ‐nBR metric showed fair to poor reliability of the dynamic parameter, and this disparity may be attributed to inaccuracies in gas switching time points, impacting on the fitting, as the gas blender was manually operated. Therefore, further investigation is necessary to optimize the enhancement (see Section 4.5 for details).

### 4.3 Density correction improves repeatability

While previous studies have demonstrated that density variation due to respiration could provide useful physiological parameters, in the current study, our motivation was to optimize the OE signal. We, therefore, utilized the adapted sponge model, which was introduced by Zha et al.^22^ to correct for density variation. In line with the previous reports,^21^, ^22^, ^23^, ^24^ our results demonstrate that the density correction significantly improves quantification of OE‐MRI metrics by decreasing fluctuation due to respiratory motion‐induced signal changes (Table 2, Figure S4). This is particularly useful in posterior slices (in supine position) where fluctuation of signal changes is greater (Figure S2).

The accuracy of the sponge model for density correction depends on the assumption that all signal change is due to density variation associated with ventilation. In practice, it is likely that other factors, such as changes in blood volume and local alveolar susceptibility profiles, also influence the signal change during the breathing cycle, and that these factors may vary depending on disease status. Nevertheless, the clear reduction in breathing‐related signal variation after correction provides evidence that the density correction is largely successful in our experiments. Furthermore, our results show that the proposed method at 3T yields excellent intra‐scanner repeatability after correction of pixel‐wise signal intensity using the deformation fields from image registration (Table 3). Previous studies utilizing a non‐Cartesian UTE approach with free‐breathing at 1.5T^22^ or breath‐held acquisitions at 0.55T^10^ similarly demonstrated improvement of repeatability in both mean PSE and the low‐enhancement percent.

### 4.4 Signal variation with position in the lung

Mean signal intensity for both baseline and O_2_‐induced change is observed to be higher in posterior slices across all subjects due to greater proton density in subject's supine position (Figures 2C,E and S2). Interestingly, we also observed that the absolute PSE of TE_1_ gradually decreases, from anterior to posterior slices, across all subjects (Figure 4A) whereas the PSE of TE_2_ does not noticeably change (Figure 4B). While PSE combines both ∆T_1_ and ∆T_2_* effects, the PSE at TE_1_ contains a stronger ∆T_1_ effect than that at TE_2_, as shown in the simulation (Figure 1D). On the contrary, ∆T_2_* effect is more substantial and dominating over ∆T_1_ effect in the PSE at TE_2_. Thus, this may be attributed to increasing effect of ∆T_1_ from anterior to posterior, possibly due to increased vessel density and/or blood pooling due to gravity, which require further investigation. A trend of ∆R_2_* increase observed from anterior to posterior slices may reflect the expected predominant sensitivity of ∆R_2_* to ventilation, as more ventilation is expected in the posterior slices when the lungs are in supine position. This is consistent with a previous report that T_2_*‐related signal is potentially more specific to ventilation due to an effect of changing concentrations of oxygen gas in the alveoli.^15^

### 4.5 Limitations and future directions

The present study has several limitations. First, we employed a 2D multi‐slice readout, which was designed to prioritize relatively high temporal resolution and allow reasonable lung coverage while accommodating free‐breathing for participant comfort. Although the temporal resolution of 1.54 s is currently the highest achievable resolution in dynamic lung OE‐MRI, it cannot still resolve all cardiac and respiratory motion‐related artifacts during acquisition. Moreover, a 2D interleaved multi‐slice excitation affects the signal variation due to through‐slice respiratory motion and inflow of blood, which are likely to be a source of noise for our T_2_*‐sensitized signal. Additionally, slice gaps and the limited number of slices may mean that some localized pathology could be missed. This could be mitigated by increasing the number of slices, either by increasing TR (thereby lowering temporal resolution and leading to more motion‐related image blurring and artifacts) or by employing acceleration methods. Since there may be inconsistencies between the slice positions, a multi‐slice acquisition also leads to challenges for inter‐scanner, inter‐session image registration that is essential for voxel‐wise comparison. The 3D non‐Cartesian UTE OE‐MRI methods have been demonstrated at 1.5T and lower field strengths to allow isotropic spatial resolution whole lung OE‐MRI measurements.^10^, ^17^, ^22^, ^31^ However, those studies are limited to static acquisitions which employed either breath‐hold or two separate free‐breathing sessions of normoxia and hyperoxia. Dynamic OE‐MRI using such methods may be possible by employing temporal view sharing methods, but we are unaware of any studies to date that have made use of this strategy. Although currently existing dynamic methods utilize respiratory gating approaches resulting in longer temporal resolution compared to our proposed method, it's worth noting that these 3D dynamic methods offer enhanced spatial resolution and SNR. Consequently, there is a need for future investigations to explore the implementation of 3D UTE acquisition in our proposed method, while maintaining a reasonable temporal resolution.

Second, our study design lacked a reference standard due to the absence of established dynamic OE‐MRI methods at 3T. This also aligns with a key motivation of the present study, which focuses on developing a reliable protocol tailored for 3T. Future investigations could compare our methods with OE‐MRI at lower field strengths or with other functional lung MRI methods.

Thirdly, low SNR in the current study leads to poor performance in extracting dynamic parameters, particularly wash‐in time. Although our results show that PSE and △R_2_* measures are repeatable, these largely reflect steady‐state conditions. Nonetheless, our primary aim was to explore a complete free‐breathing acquisition approach that spans the entire gas delivery time course for both air and O_2_ phase. This approach not only enhances subject comfort but also maintains physiological realism, features that breath‐hold or separate free‐breathing methods for each gas phase may lack. Therefore, the feasibility of the proposed method with the full free‐breathing acquisition suggests potential for future development of OE‐MRI for evaluating dynamic parameters. Furthermore, optimizing the methodological approach, such as new hardware for the administration or additional monitoring to track breathing patterns, might improve reproducibility in this measurement.

Lastly, being a proof‐of‐concept investigation, our current study is limited by a small sample size and the absence of individuals with disease. Future studies will provide a more comprehensive understanding of the method's applicability in such settings.

## 5 CONCLUSIONS

Our study establishes the viability of dynamic lung OE‐MRI at 3T, optimizing a dual‐echo RF‐spoiled gradient echo acquisition for simultaneous PSE, R_2_* changes, and oxygen wash‐in measurement during free‐breathing, offering functional information. Excellent intra‐scanner repeatability and good inter‐scanner reproducibility of the metrics suggest multi‐center clinical application will be feasible. Future studies in respiratory diseases may allow us to better understand the method's potential.

## CONFLICT OF INTEREST STATEMENT

G.J.M. Parker is an employee of and holds ownership interest (including patents) in Bioxydyn Limited. J. Naish and M. Tibiletti are employees of Bioxydyn Limited. No potential conflicts of interest were disclosed by the other authors.

## Supporting information


**Table S1.** Simulation parameters.
**Table S2.** Shapiro–Wilk normality test before and after log‐transformation of τ‐nBR values. The *p*‐values below 0.05 suggests that the data significantly deviates from a normal distribution.
**Figure S1.** A workflow diagram summarizing the experimental study population and data analysis.
**Figure S2.** Example time course curves of the median signal intensity (SI) and R_2_* from masked, registered lung for each slice of a single traveling subject obtained in London (A) and Manchester (B) with TE_1L_ (0.71 ms), TE_2L_ (1.2 ms), TE_1M_ (0.81 ms), and TE_2M_ (1.51 ms), by pre‐ (blue line) and post‐tissue density correction (red line).
**Figure S3.** (A) Pre‐ and (B) post‐density corrected example time course (blue dashed lines) and fits (red solid lines) for downslopes and upslopes from an individual voxel.
**Figure S4.** The Bland–Altman plots for the repeated measurements of percent signal change (PSE) averaged over two posterior slices from the 1st and 2nd TE before (A, C for TE_1L_ and TE_2L_, respectively) and after tissue density correction (B, D for TE_1L_ and TE_2L_, respectively). The 95% LOA decreased from (−7.22%, 4.55%) to (−2.36%, 1.33%) for TE_1L_ and (−7.53%, 6.20%) to (−2.99%, 1.84%) for TE_2L_. Similarly, additional statistical metrics display significantly reduced RC (69% and 65% for TE_1L_ and TE_2L_, respectively) and increased ICC_intra_ (94% and 75% for TE_1L_ and TE_2L_, respectively) with tissue density correction compared to pre‐density correction.
**Figure S5.** Bland–Altman analysis plots illustrating the repeated measurements of wash‐in time normalized for breathing rate (non‐transformed breathing rate [τ‐nBR]; unitless) representing the breath count during τ, for the 1st and 2nd TE. The data points correspond to individual participants for inter‐scanner difference between two visits to Manchester and London sites (A, B) and intra‐scanner difference between two scans in London site (C, D). See also Table 3.
**Figure S6.** Simulated versus experimental percent signal change (PSE) values plotted as a function of TE. For the simulation, we utilized literature‐reported values for T_1_ (1281 ms for air and 1102 ms for 100% O_2_) and incorporated measured T_2_* values from the lungs at air and 100% O_2_ breathing, acquired using the same experimental protocol as detailed in this study (Table S1). For each traveling volunteer, we averaged T_2_* values across two sites (London and Manchester) from two posterior slices. These averaged T_2_* values for eight volunteers listed on the right‐hand side (as displayed in Table S1 of the supporting information) were used to simulate eight individual PSE plots. The experimental PSE values were obtained at four separate echo times (TE_1L_ = 0.71 ms, TE_2L_ = 1.2 ms in London and TE_1M_ = 0.81 ms, TE_2M_ = 1.51 ms in Manchester) and averaged across multiple either (A) two posterior or (B) all six slices for each traveling volunteer. The collective PSE results from the two MRI systems exhibit a comparable trend in relation to the TE while the simulated PSE plots show variability influenced by individual T_2_* values.
