# Supplementary material for: Feasibility of dynamic T2 *‐based oxygen‐enhanced lung MRI at 3T
Source: Magn Reson Med. 2023 Nov 27;91(3):972–86. doi: 10.1002/mrm.29914 (PMC10952203; doi:10.1002/mrm.29914)

**Supporting Information**

Table S1. Simulation parameters

| Parameters used for all simulations (Fig.1 and Fig.S6) | | | | | | | | |
| --- | --- | --- | --- | --- | --- | --- | --- | --- |
| TR (ms) | 16 | | | | | | | |
| Flip Angle (°) | 5 | | | | | | | |
| T_1_ air (ms) | 1281 | | | | | | | |
| T_1_ 100% O_2_ (ms) | 1102 | | | | | | | |
| Parameters used for Fig.1: T_2_* averaged across 32 data sets | | | | | | | | |
| T_2_* air (ms) | 0.68 | | | | | | | |
| T_2_* 100% O_2_ (ms) | 0.62 | | | | | | | |
| Parameters used for supporting information Fig.S6: T_2_* averaged across two sites for each travelling volunteer (TV) | | | | | | | | |
|  | TV1 | TV2 | TV3 | TV4 | TV5 | TV6 | TV7 | TV8 |
| T_2_* air (ms) | 0.75 | 0.67 | 0.69 | 0.74 | 0.70 | 0.68 | 0.75 | 0.65 |
| T_2_* 100% O_2_ (ms) | 0.67 | 0.62 | 0.63 | 0.69 | 0.63 | 0.62 | 0.69 | 0.60 |

Table S2. Shapiro-Wilk normality test before and after log-transformation of τ-nBR values. The p-values below 0.05 suggests that the data significantly deviates from a normal distribution.

|  |  | Non-transformation | Log-transformation |
| --- | --- | --- | --- |
| TE_1_ | Manchester | 0.021 (non-normal) | 0.084 |
|  | London | 0.252 | 0.492 |
|  | London1 | 0.160 | 0.846 |
|  | London2 | 0.018 (non-normal) | 0.447 |
| TE_2_ | Manchester | 0.548 | 0.899 |
|  | London | 0.789 | 0.624 |
|  | London1 | 0.594 | 0.970 |
|  | London2 | 0.001 (non-normal) | 0.119 |

Figure S1. A workflow diagram summarising the experimental study population and data analysis.


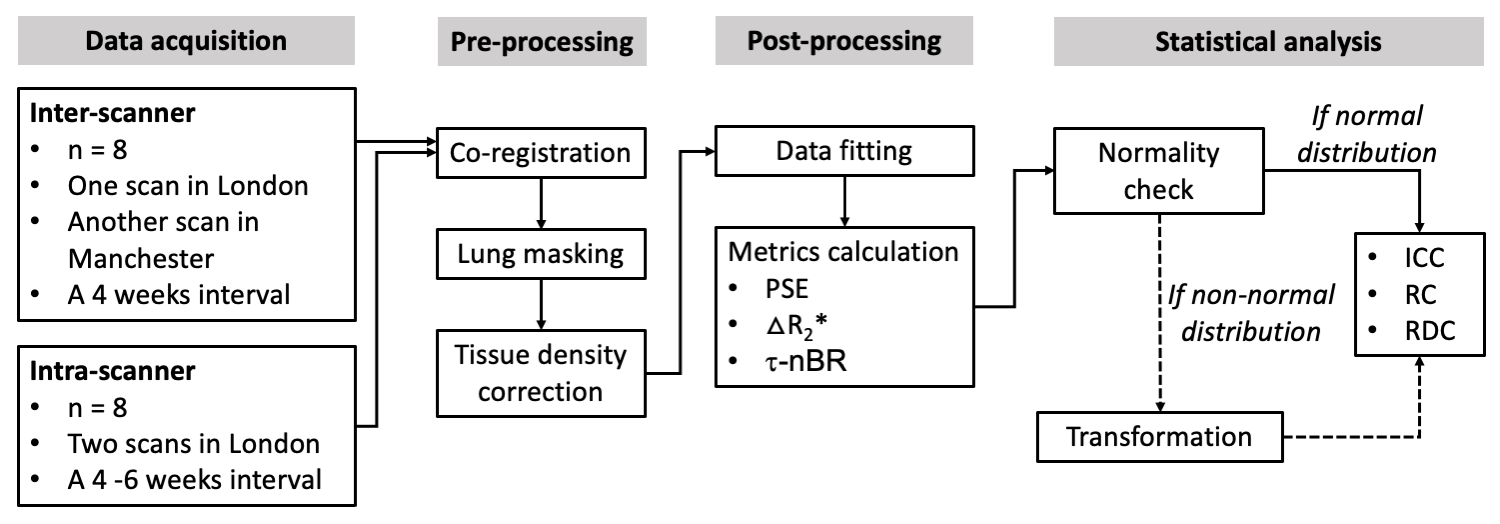


Abbreviations: PSE (Percent signal enhancement); τ-nBR (wash-in time normalised for breathing rate); ICC (Intra-class correlation coefficient); RC (repeatability coefficient); RDC (reproducibility coefficient)

Figure S2. Example time course curves of the median signal intensity (SI) and R_2_* from masked, registered lung for each slice of a single travelling subject obtained in London (A) and Manchester (B) with TE_1L_ (0.71 ms), TE_2L_ (1.2 ms), TE_1M_ (0.81 ms), and TE_2M_ (1.51 ms), by pre- (blue line) and post-tissue density correction (red line).


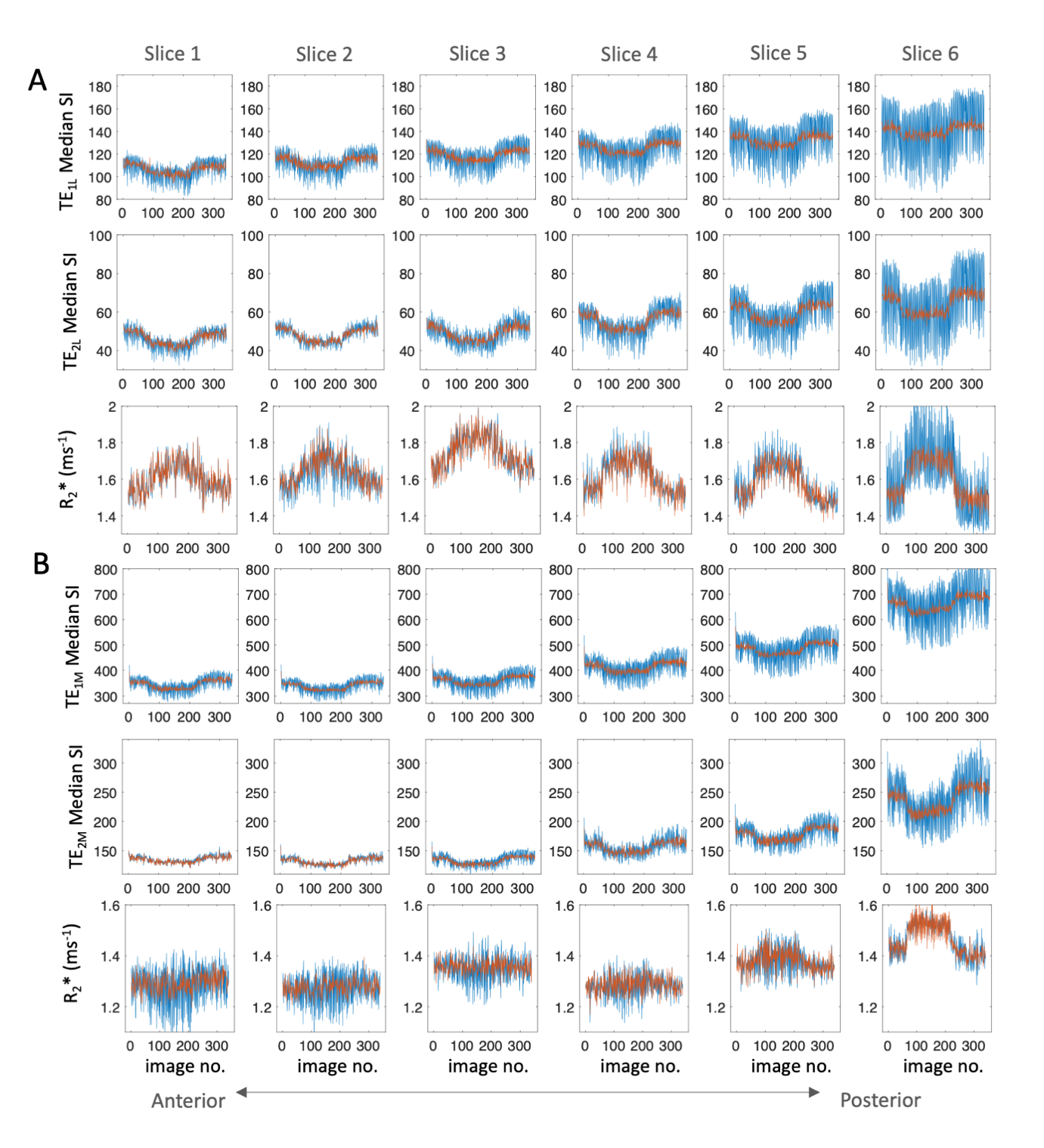


Figure S3. (A) Pre- and (B) post-density corrected example time course (blue dashed lines) and fits (red solid lines) for downslopes and upslopes from an individual voxel.


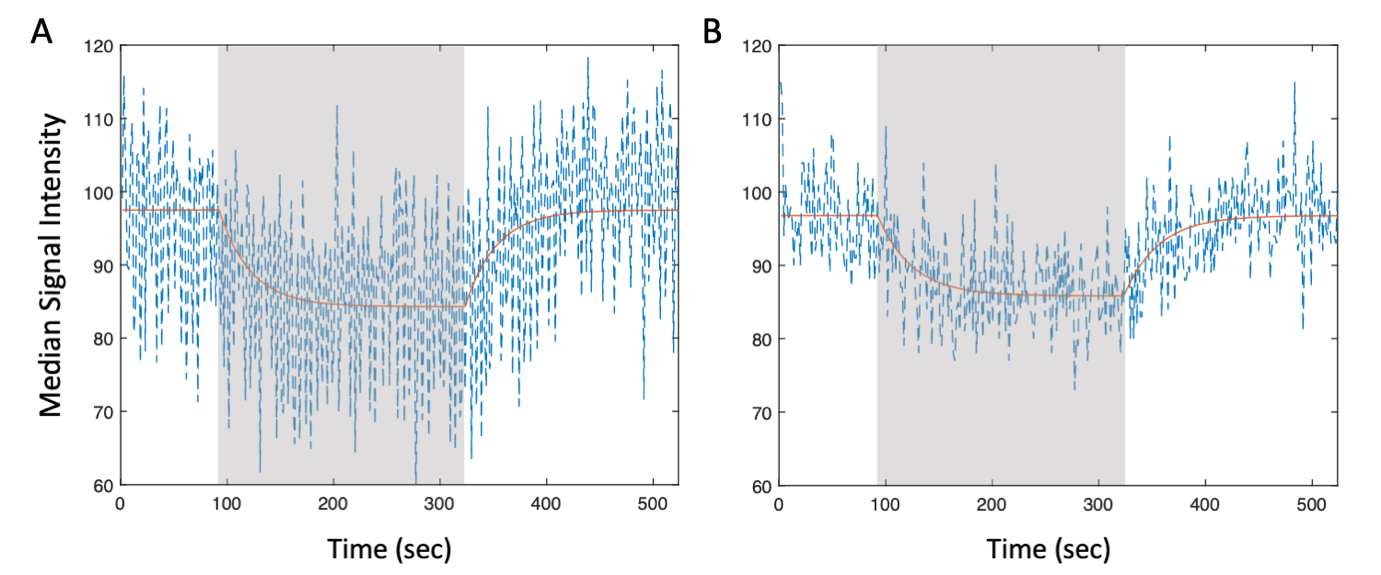


Figure S4. The Bland-Altman plots for the repeated measurements of percent signal change (PSE) averaged over two posterior slices from the 1^st^ and 2^nd^ TE before (A and C for TE_1L_ and TE_2L_, respectively) and after tissue density correction (B and D for TE_1L_ and TE_2L_, respectively). The 95% LOA decreased from [-7.22%, 4.55%] to [-2.36%, 1.33%] for TE_1L_ and [-7.53%, 6.20%] to [-2.99%, 1.84%] for TE_2L_. Similarly, additional statistical metrics display significantly reduced RC (69% and 65% for TE_1L_ and TE_2L_, respectively) and increased ICC_intra_ (94% and 75% for TE_1L_ and TE_2L_, respectively) with tissue density correction compared to pre-density correction.


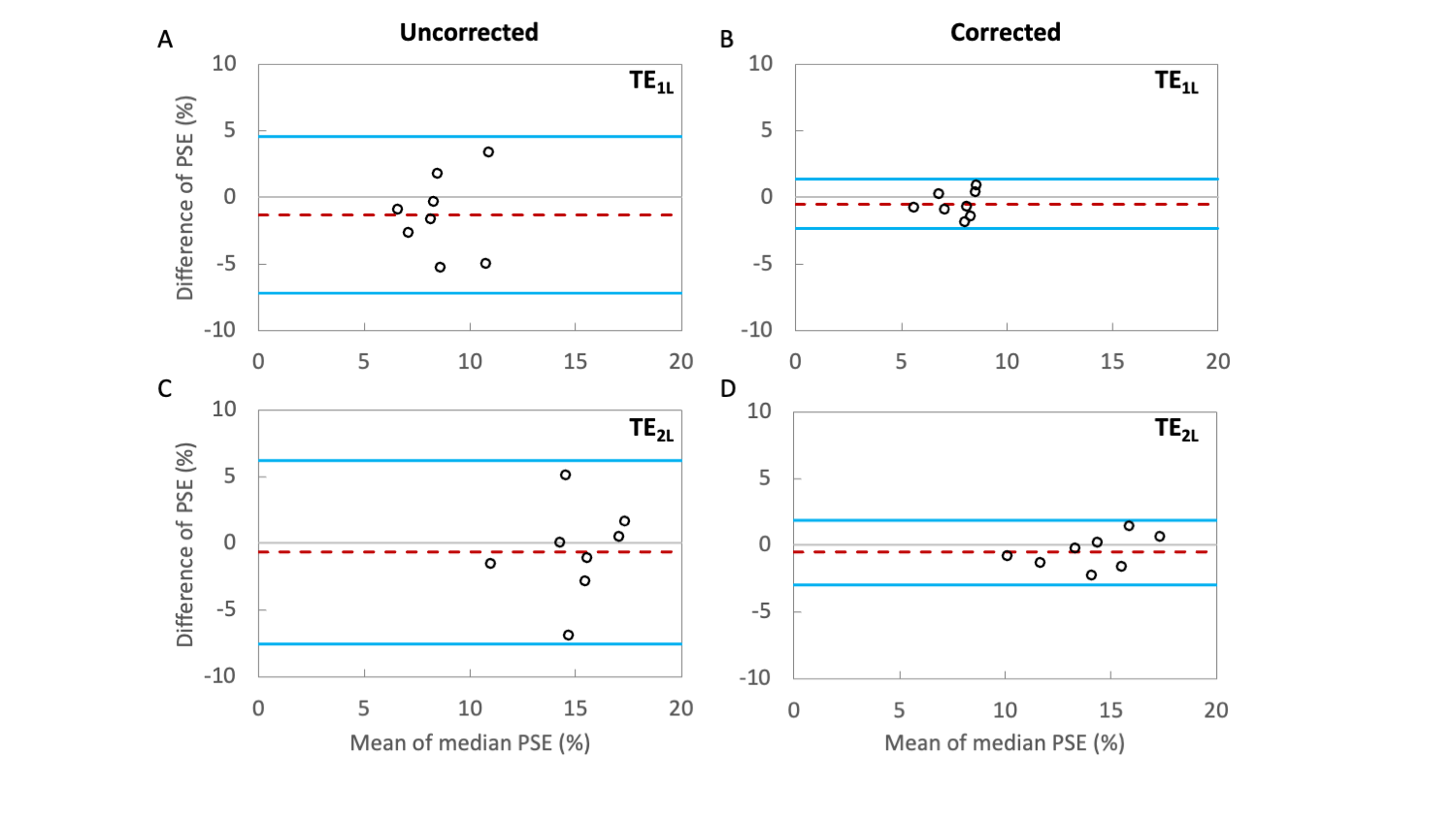


Figure S5. Bland-Altman analysis plots illustrating the repeated measurements of wash-in time normalised for breathing rate (non-transformed τ-nBR; unitless) representing the breath count during wash-in time, for the 1^st^ and 2^nd^ TE. The data points correspond to individual participants for inter-scanner difference between two visits to Manchester and London sites (A and B) and intra-scanner difference between two scans in London site (C and D). See also Table 3.


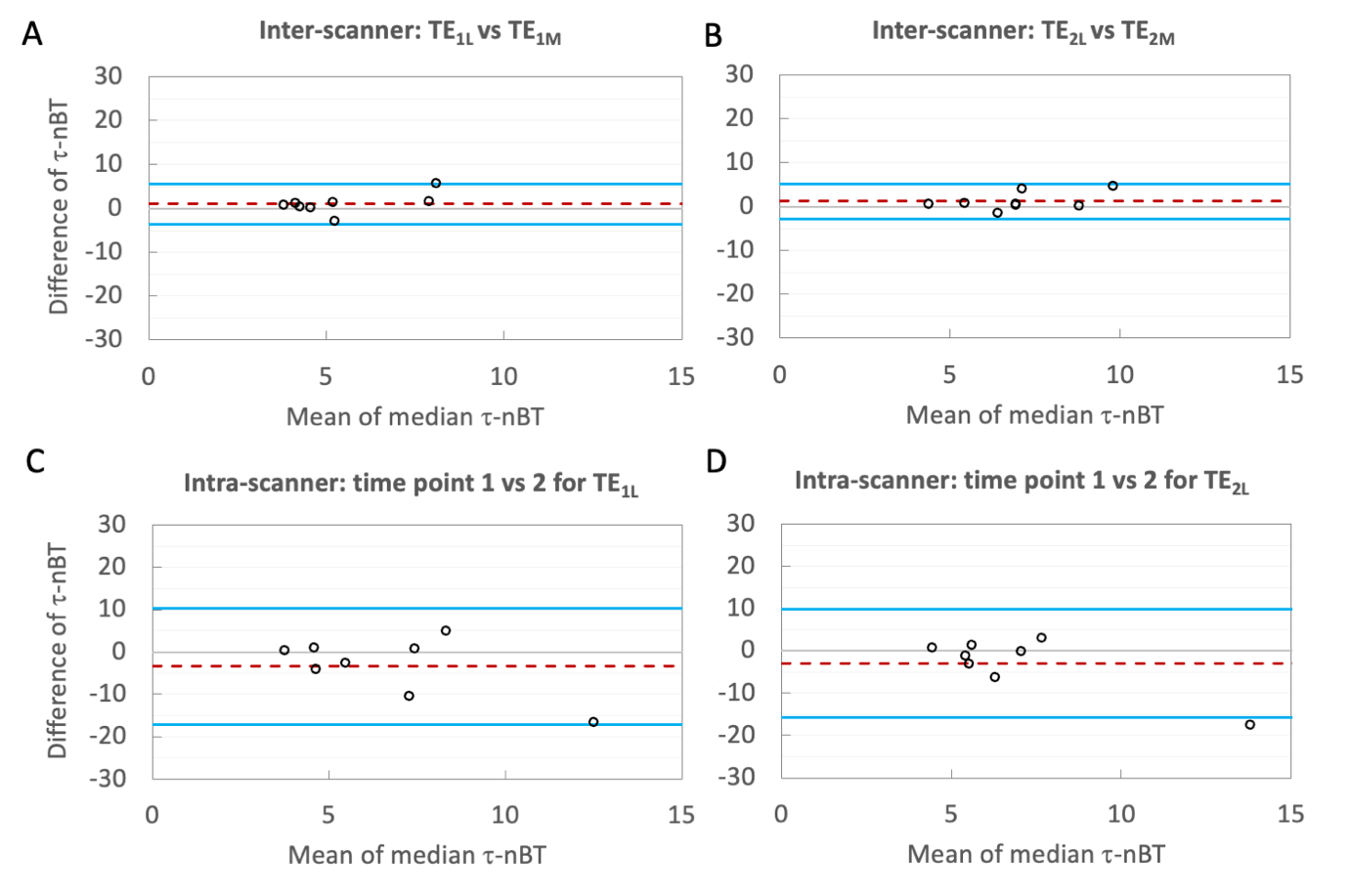


Figure S6. Simulated vs experimental percent signal change (PSE) values plotted as a function of TE. For the simulation, we utilised literature-reported values for T_1_ (1281 ms for air and 1102 ms for 100% O_2_) and incorporated measured T_2_* values from the lungs at air and 100% O_2_ breathing, acquired using the same experimental protocol as detailed in this study (Table S1). For each travelling volunteer, we averaged T_2_* values across two sites (London and Manchester) from two posterior slices. These averaged T_2_* values for eight volunteers listed on the right-hand side (as displayed in Table S1 of the supporting information) were used to simulate eight individual PSE plots. The experimental PSE values were obtained at four separate echo times (TE_1L_ = 0.71 ms, TE_2L_ = 1.2 ms in London and TE_1M_ = 0.81 ms, TE_2M_ = 1.51 ms in Manchester) and averaged across multiple either (A) two posterior or (B) all six slices for each travelling volunteer. The collective PSE results from the two MRI systems exhibit a comparable trend in relation to the TE while the simulated PSE plots show variability influenced by individual T_2_* values.


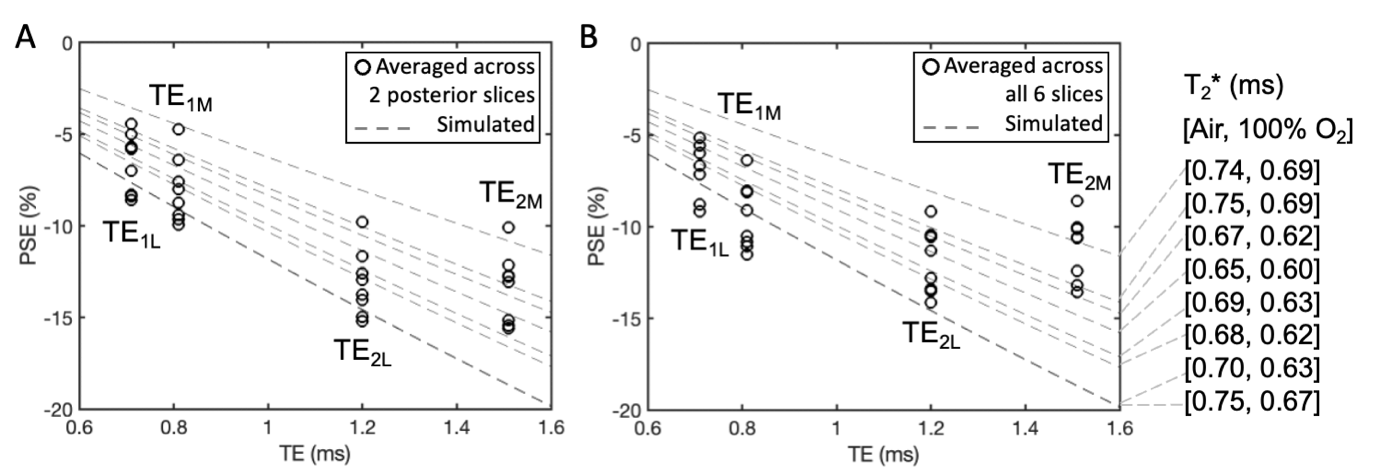

Supplement: Supplementary file 1 — Table S1. Simulation parameters. Table S2. Shapiro–Wilk normality test before and after log‐transformation of τ‐nBR values. The p‐values below 0.05 suggests that the data significantly deviates from a normal distribution. Figure S1. A workflow diagram summarizing the experimental study population and data analysis. Figure S2. Example time course curves of the median signal intensity (SI) and R2* from masked, registered lung for each slice of a single traveling subject obtained in London (A) and Manchester (B) with TE1L (0.71 ms), TE2L (1.2 ms), TE1M (0.81 ms), and TE2M (1.51 ms), by pre‐ (blue line) and post‐tissue density correction (red line). Figure S3. (A) Pre‐ and (B) post‐density corrected example time course (blue dashed lines) and fits (red solid lines) for downslopes and upslopes from an individual voxel. Figure S4. The Bland–Altman plots for the repeated measurements of percent signal change (PSE) averaged over two posterior slices from the 1st and 2nd TE before (A, C for TE1L and TE2L, respectively) and after tissue density correction (B, D for TE1L and TE2L, respectively). The 95% LOA decreased from (−7.22%, 4.55%) to (−2.36%, 1.33%) for TE1L and (−7.53%, 6.20%) to (−2.99%, 1.84%) for TE2L. Similarly, additional statistical metrics display significantly reduced RC (69% and 65% for TE1L and TE2L, respectively) and increased ICCintra (94% and 75% for TE1L and TE2L, respectively) with tissue density correction compared to pre‐density correction. Figure S5. Bland–Altman analysis plots illustrating the repeated measurements of wash‐in time normalized for breathing rate (non‐transformed breathing rate [τ‐nBR]; unitless) representing the breath count during τ, for the 1st and 2nd TE. The data points correspond to individual participants for inter‐scanner difference between two visits to Manchester and London sites (A, B) and intra‐scanner difference between two scans in London site (C, D). See also Table 3. Figure S6. Simulated versus experimental percent signa [file MRM-91-972-s001.docx]
